# Gengnianchun Recipe Protects Ovarian Reserve of Rats Treated by 4-Vinylcyclohexene Diepoxide via the AKT Pathway

**DOI:** 10.1155/2020/9725898

**Published:** 2020-12-16

**Authors:** Fangui Zhao, Wenjun Wang

**Affiliations:** ^1^Department of Ultrasound Diagnosis, Obstetrics and Gynecology Hospital, Fudan University, Shanghai 200011, China; ^2^Shanghai Key Laboratory of Female Reproductive Endocrine Related Diseases, Shanghai 200011, China; ^3^Department of Integrated Traditional Chinese Medicine and Western Medicine, Obstetrics and Gynecology Hospital, Fudan University, Shanghai 200011, China

## Abstract

Diminished ovarian reserve (DOR) refers to a decrease in the number and quality of oocytes. Western treatment of DOR does not improve the ovarian reserve fundamentally, and the effect is limited. Gengnianchun recipe (GNC) is a traditional Chinese medicine formula originally applied to treat menopausal syndrome but is also found to be effective in treating clinical DOR patients. Here we aim to examine the effect of GNC in a DOR rat model induced by 4-vinylcyclohexene diepoxide (VCD), a chemical that selectively destroys ovarian small preantral follicles, and further investigate the possible mechanisms. Female SD rats were randomly divided into four groups: control group (C), model group (M), high-dose GNC group (H), and low-dose GNC group (L). Rats in M, H, and L were administered with VCD and normal saline, high-dose GNC, and low-dose GNC separately. Rat ovaries were harvested either to conduct HE staining for follicle count, immunohistochemistry, or western blot. We found that high dose of GNC significantly increased the ovarian index and sustained the number of primordial follicles and primary follicles in VCD treated rats. Moreover, high dose of GNC significantly increased the ovarian protein expression of mouse vasa homologue (MVH), anti-Müllerian hormone (AMH), follicle-stimulating hormone receptor (FSHR), and estrogen receptor *β* (ER*β*) compared with that in the model group. Besides, high-dose GNC significantly increased ovarian AKT phosphorylation and the expression of downstream forkhead box O3 (FOXO3a). Proapoptosis proteins of Bax, cleaved caspase-3, and poly ADP-ribose polymerase (PARP) were significantly decreased after high-dose GNC treatment compared with those in the model group. Taken together, these findings suggest that high-dose GNC could protect ovarian reserve against VCD-induced toxicity via the activation of the AKT signaling pathway and reduced cell apoptosis in SD Rats. This effect could either be induced by the increased FSHR signaling or by the nontranscriptional activation of ER*β*, which requires further investigation.

## 1. Introduction

Diminished ovarian reserve (DOR) refers to a decrease in the number and quality of oocytes [1, 2]. DOR patients may have irregular or regular menstruation but bear a lower chance of pregnancy and a higher chance of abortion. Different from the physiological ovarian reserve decline after menopause, DOR usually refers to the early or accelerated occurrence of ovarian reserve decline in women of reproductive age [3]. The etiology of DOR remains unclear. Genetic factors, environmental pollution, autoimmune diseases, psychological factors, and iatrogenic factors may be involved. Current consensus holds that it is a disease with complex causes and lack of treatment approaches with definitive efficacy.

Gengnianchun recipe (GNC) is an effective traditional Chinese formula applied in our hospital to treat postmenopausal syndrome for decades [4]. Our group has studied the effect of GNC on various animal models to reveal its mechanism in treating various aging-related diseases such as osteoporosis, Alzheimer's disease, immune dysfunction, and skin aging [5–13]. In traditional Chinese medicine theories, DOR shares a similar etiology with menopause, which is the deficiency in the kidney. Therefore, we have also applied GNC on clinical DOR patients and observed great effect in recovering ovarian reserve and improving pregnancy rate. But this has not been verified in the animal model and the mechanism remains unknown.

4-vinylcyclohexene diepoxide (VCD) is an environmental chemical produced during the manufacture of rubber tires, flame retardants, insecticides, plasticizers, and antioxidants. Earlier studies have shown that short-term use of VCD can damage the primordial follicles and primary follicles of Sprague-Dawley (SD) rat ovaries to induce the initial stage of DOR [14, 15]. Also, VCD might work through the inhibition of the PI3K-AKT signaling pathway to induce apoptosis and follicle atresia [16, 17]. In this study, we aim to explore the effect of GNC on a VCD-induced DOR rat model and to further investigate its influence on PI3K-AKT pathway molecules to explore the underlying mechanism, thereby providing an experimental basis for the application of GNC in DOR therapy.

## 2. Materials and Methods

### 2.1. Animals and Treatments

A total of forty 3- to 4-week-old female SD rats weighing 35 ± 3.5 g were purchased from Shanghai JieSiJie Laboratory Animal Co., Ltd., China. Rats were housed in the SPF facility with a standard pellet diet and water was provided ad libitum at a temperature of 22–25°C and relative humidity of 50–60% with daily 12-hour light. After adaptation for 5 days, all rats were randomly divided into four groups: control group (C), model group (M), high-dose GNC group (H), and low-dose GNC group (L). Rats in group M, H, and L were given VCD (94956, Sigma-Aldrich, USA) by intraperitoneal injection on a dosage of 80 mg/kg bodyweight for 20 days according to previous reports [15, 18], while rats in group C were administered the same volume of solvent (S3547, Sigma-Aldrich, USA). In the meantime, rats in the groups M, H, and L were given normal saline, high-dose (5.54 g/kg body weight), or low-dose (2.77 g/kg body weight) of GNC decoction, respectively, by gavage for 30 days. The low dose was converted from the human dose according to a previous report [19]. High dose was twice as high as the low dose. The time length of GNC application was set according to the clinical application length on DOR patients, which is 6 menstruation cycles and equals 30 days on rats. The GNC decoction was prepared by dissolving herb granules into hot water [7]. Intraperitoneal injection and oral gavage volume were adjusted according to the bodyweight, which was measured every three days [13]. All animal experimental procedures were approved by the Animal Experimental Ethical Committee of Fudan University (no.2015-07-FCKYY-WWJ-01).

### 2.2. Sample Collection

After 30 days of manipulation, rats were given vaginal smears and observed for the predominant cell type under a light microscopic to verify the estrous cycle stage [20]. Rats that were not at estrous stage were selected to be sacrificed. Other rats were continued with previous manipulation and sacrificed until vaginal smear results turned to other stages of estrous cycle. Whole blood was collected from the heart after anesthesia. The collected whole blood was centrifuged at 3000 rpm for 10 minutes at 4°C to retrieve serum. Bilateral ovaries were collected and immediately weighed to calculate the ovarian index by the following formulation: ovarian index = ovarian wet weight/body weight. Uteruses were also collected for morphology observation. The left ovaries were fixed in 4% paraformaldehyde solution for 24 hours and then embedded in paraffin for preparation of slides by Wuhan Servicebio Technology Co., Ltd. The right ovaries were dipped in liquid nitrogen and transferred to a-80°C freezer until further use.

### 2.3. Ovary Serial Section and Follicle Counting

After the ovarian tissue was paraffin-embedded, the largest sections of ovaries were selected for continuous sectioning. Three sections at the 10th, 20th, and 30th sections of the largest sections of each ovary were selected to perform hematoxylin-eosin (HE) staining for histological observation and follicle count. Follicles were classified into various stages according to the modified Oktay system [21]. The analysis was repeated three times with each replicate containing 18 slices of 6 ovaries from 6 rats in each group.

### 2.4. Serum Hormone Assay

Serum estradiol (*E*_2_) and testosterone (*T*) levels were detected by the chemiluminescence method. The commercially available kits were used to measure the serum concentration of *E*_2_ (33540, Beckman, USA, detection range: 13–4096pg/mL) and *T* (33550, Beckman, USA, detection range: 0.1–16 ng/mL) by the Beckman Coulter Unicel DxI 800 system according to the manufacturer's protocols. The analysis was repeated three times with each replicate containing 6 samples from 6 rats in each group.

### 2.5. Immunohistochemistry

The ovarian tissue sections were dewaxed with xylene and ethanol. After the antigen retrieval, the sections were exposed to primary antibodies overnight at 4°C. Antibody information has been listed in Table 1. The tissues were incubated with horseradish peroxidase-linked secondary antibodies for 40 minutes at room temperature and then stained with DAB solution and hematoxylin for visualization. After dehydration, the sections were sealed with neutral gum and observed under a microscope. The analysis was repeated three times with each replicate containing 6 slices of 6 ovaries from 6 rats in each group.

### 2.6. Western Blot

The right ovaries were dipped in the lysis buffer and homogenized by an ultrasound homogenizer. The lysate was then centrifuged at 12000 rpm for 30 minutes at 4°C. The supernatant was harvested and boiled with loading buffer for SDS-PAGE. Proteins were subjected to electrophoresis in SDS-polyacrylamide gels and transferred to polyvinylidene fluoride membranes. After blocking with nonfat milk, the membranes were exposed to primary antibodies overnight at 4°C. Antibody information has also been listed in Table 1. Afterward, the membrane was incubated with antirabbit IgG HRP-linked antibodies (#7074, CST, Inc., USA) for 1 h at room temperature and then visualized by the chemiluminescence method and observed under an ImageQuant LAS-4000 mini biomolecular imager, GE, USA. The relative protein expression levels were determined by Quantity One software. The analysis was repeated three times with each replicate containing 6 slices of 6 ovaries from 6 rats in each group.

## 3. Statistical Methods

The experimental data were presented as mean ± standard deviation (*X* ± SD). SPSS18.0 was used for statistical analysis. The comparison of body weight changes between four groups was made using repeated measurement analysis of variance. ANOVA with the Bonferroni post hoc test was applied to test significances between treatment groups. *P* < 0.05 was considered as a significant difference.

## 4. Results

### 4.1. General Condition of Rats and Observation of Reproductive Organs

**Figure 1:**
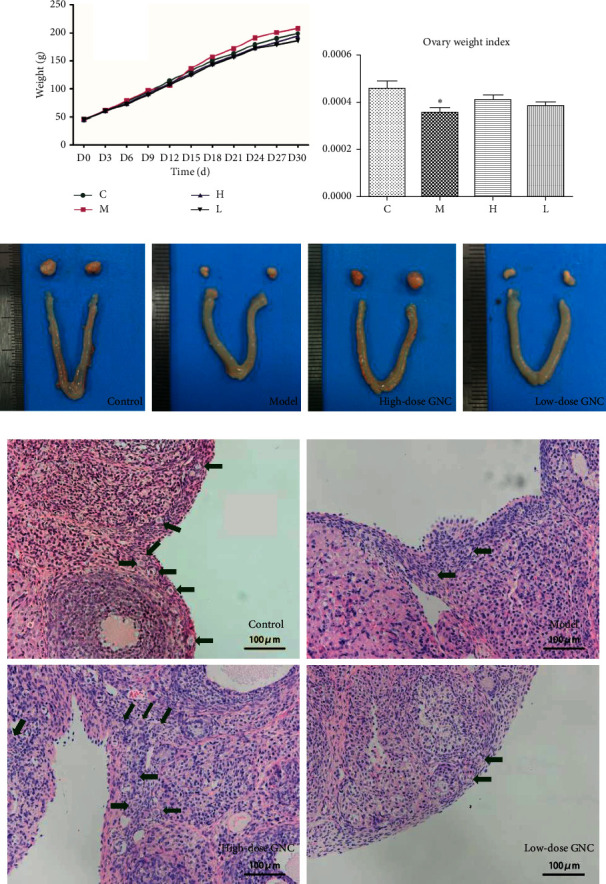
General conditions of rats and reproductive organs in different groups. (a) Bodyweight changes of rats in each group during experimental manipulation. (b) Ovarian weight index of rats in each group. (c) The representative appearance of ovaries and the uterus of rats in each group. (d) Representative view of HE staining of ovaries under a microscope. The brown arrows indicate primordial follicles.

To adjust the dose of VCD and GNC, body weight changes of rats in different groups were monitored every three days during 30-day manipulation. As Figure 1(a) shows, there was no significant difference between the groups during drug administration. This indicates that VCD and GNC do not affect the general health of rats. After 20 days of VCD administration, the ovarian index of the model group was significantly decreased compared to the control group (Figure 1(b), *p* < 0.05). After high-dose GNC treatment, the mean ovarian index seemed to increase a little than the group M. But the difference between the group M and group H was not significant. Low dose of GNC did not affect the ovarian index. The appearance of the uterus and ovaries in the different groups is shown in Figure 1(c). We observed a lower volume of ovaries in the group M and L compared to that of the group C and H. Also, there seemed to be less big antral follicles in the ovarian cortex in the group M and L than that of the group C and H. Representative microscopic view of ovarian HE staining shows that there were plenty of primordial follicles accumulate at the ovarian cortex in the group C and H, while the number of primordial follicles in the cortex in the group M and L was much lower (Figure 1(d)).

### 4.2. Ovarian Reserve Changes after VCD and GNC Treatment

**Figure 2:**
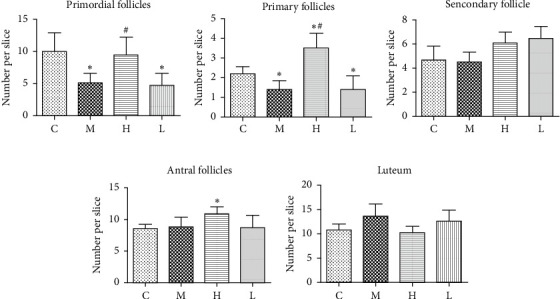
The number of follicles at different stages (a–d) and the number of corpus luteum (e) in the ovaries of rats in each group. ^∗^: significant compared with group (C) *p* < 0.05; #: significant compared with group (M) *p* < 0.05.

As per the previous studies report, VCD mainly induced a significant decline in primordial and primary follicles compared to the control group (Figures 2(a) and 2(b), *P* < 0.05). High dose of GNC treatment significantly improved the number of primordial follicles and primary follicles (Figures 2(a) and 2(b), *P* < 0.05). The mean number of primary follicles was even higher than the control group (Figure 2(b), *P* < 0.05). There was no significant difference between the group M and C in secondary follicles, antral follicles, or corpus luteum (Figures 2(c)–2(e)). Also, there was no therapeutic effect of low-dose GNC observed in improving numbers of follicles at all stages or corpus luteum compared to the model group (Figures 2(a)–2(e)) Interestingly, high-dose of GNC significantly improved the antral follicle counts compared to both group M and group C (Figure 2(d), *P* < 0.05).

### 4.3. The Effect of VCD and GNC on the Reproductive Hormone Level

**Figure 3:**
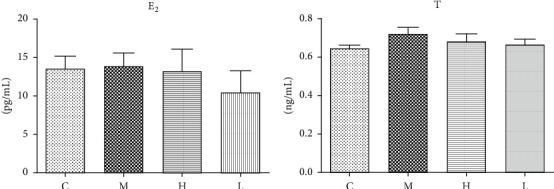
Serum *E*_2_ (a) and T (b) levels among rats in different groups.

*E*_2_ and *T* are two reproductive hormones secreted by granulosa cells and theca cells of follicles, respectively. Probably because VCD mainly causes the depletion of primordial follicles and primary follicles, and the number of both granulosa cells and theca cells are relatively small compared to larger follicles; we did not observe any difference in the *E*_2_ and *T* level between each group (Figure 3).

### 4.4. Effects of GNC on the Expression of Biomarkers of Ovarian Reserve and Hormone Receptors in the Ovary

**Figure 4:**
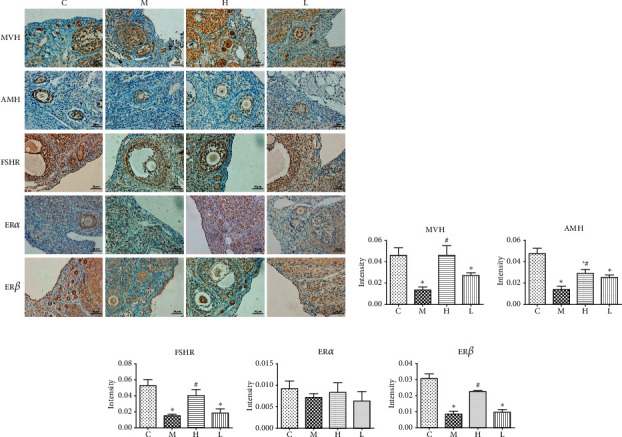
Immunohistochemical analysis of the expression of various biomarkers for ovarian reserve in each group. ^∗^: significant compared with group (C) (*p* < 0.05); #: significant compared with group (M) (*p* < 0.05).

To better verify the effect of GNC on the improvement of ovarian reserve, we performed IHC and WB assays to determine the protein expression levels of several biomarkers of ovarian reserve. MVH is expressed in the cytoplasm of the oocyte in follicles at various stages and was mainly expressed in primordial follicles and primary follicles in this experiment. AMH is mainly expressed in the cytoplasm of secondary follicles and the granulosa cells of early antral follicles. As shown in Figures 4(a)–4(c), there were more deeply stained MVH and AMH positive follicles in group C and H than those in groups M and L (*P* < 0.05). The intensity of DAB staining in primordial follicles in group L was slightly higher than that in group M, and the differences were not significant. FSHR is the receptor of FSH and intermediated the promotion effects of FSH on follicle development. Similar results with MVH and AMH were found in FSHR expression in small and large secondary follicles and antral follicles, where FSHR was mainly expressed (Figures 4(a) and 4(d)). The group C and group H had higher expression of FSHR than that in the group *M*(*P* < 0.05). The group L showed less difference compared with the group M . Estrogen is another important regulatory factor in follicle development. ER*α* is mainly expressed in the nucleus of oocytes in follicles at various stages and stromal cells, while ER*β* is mainly expressed in the cytoplasm of oocytes in follicles at various stages, especially in primordial follicles, primary follicles, and secondary follicles, with no expression in the nucleus [22]. There was no significant difference in the ER*α* expression level among follicles of ovaries at different stages in any group by immunohistochemistry (Figure 4(e)). But VCD induced a significantly downregulated ER*β* expression in ovaries (Figure 4(f), (*P* < 0.05)). Also, high-dose of GNC significantly recovered the expression level of ER*β* compared to the model group. There was also no significant effect of low-dose of GNC observed in regulating ER*β* expression.

**Figure 5:**
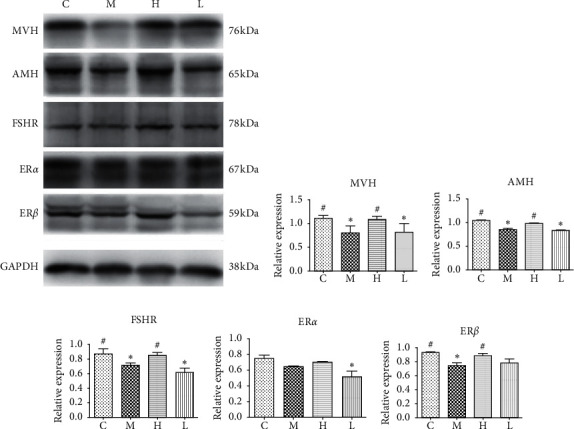
Western blot analysis of ovarian reserve function-related protein (MVH, AMH, and FSHR) and ER subtypes (ER*α* and ER*β*) in ovarian follicles of each group. ^∗^: significant compared to group (C) (*P* < 0.05); #: significant compared to the group (M) (*P* < 0.05).

Western blot analysis confirmed most of the results of immunohistochemistry. As shown in Figures 5(a)–5(d), the expression of MVH, AMH, and FSHR protein in the group M was significantly lower than that in the group C. The expression of MVH, AMH, and FSHR proteins was higher in the group H than that in the group M and was similar to that in the group C. The expression levels of MVH, AMH, and FSHR in the group L were also slightly increased or remained relatively low compared to the group M. Although group L showed a decreased ER*α* compared to the group C, there was no significant difference between the groups M, H, and L (Figure 5(e)). ER*β* showed similar trends between different groups as MVH, AMH, and FSHR. High-dose of GNC improved ER*β* expression to a relatively normal level than the group M. Also, the group L tended to have a higher level of ER*β* expression than that in the group *M*, but the difference was not significant (Figure 5(f)).

### 4.5. Effects of GNC on the PI3K-AKT Signaling Pathway

**Figure 6:**
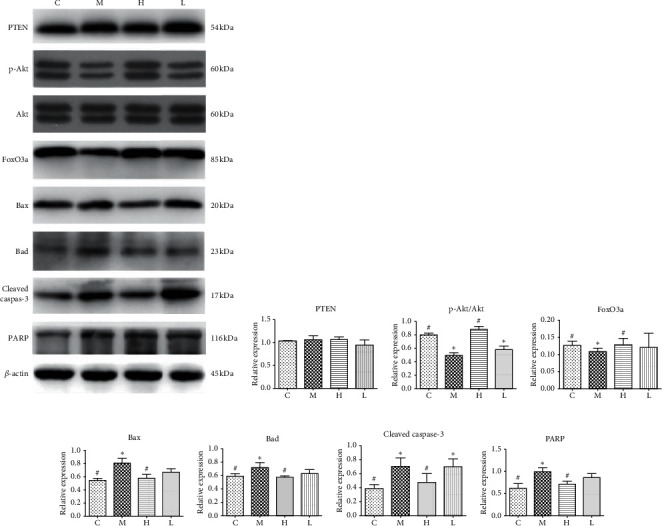
Western blot analysis of the protein expression of AKT signaling molecules in rat ovaries of each group. ^∗^: significant compared to group (C) (*P* < 0.05); #: significant compared to group (M) (*P* < 0.05).

A previous report claimed that the inhibition of the PI3K-AKT pathway intermediated the adverse effect of VCD on primordial and primary follicle atresia [17]. Therefore, we further explored the effect of GNC on the expression of upstream and key regulators of the PI3K-AKT signaling pathway and downstream apoptosis-related targets through western blot and immunohistochemistry. As shown in Figures 6(a) and 6(b), there was no significant difference in PTEN expression in ovarian tissues among different groups. Among the downstream signal molecules, the relative protein expression levels of p-AKT/AKT and FoxO3a in the group M were significantly decreased compared to that of the group C. Also, the group H showed a significantly higher expression of these proteins than that in the group M. The difference between the group L and group M was not significant (Figures 6(a)–6(d)). Besides, the expression of the proapoptotic proteins, Bad, Bax, cleaved caspase-3, and PARP was significantly increased in the group M compared to the group C. Compared with the group M, the levels of the above proapoptotic proteins in the group H were significantly decreased (Figures 6(e)–6(h)).

**Figure 7:**
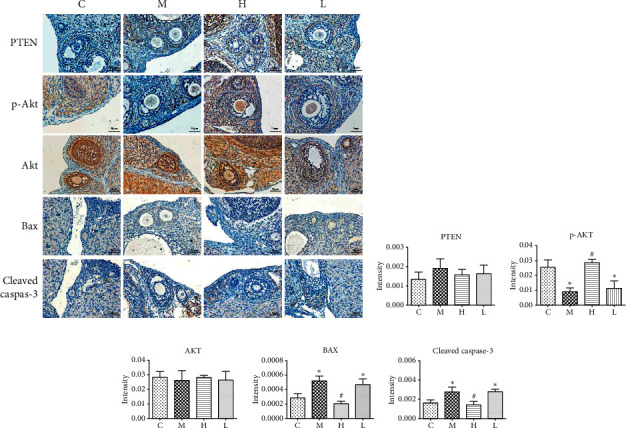
Immunohistochemistry analysis of AKT signaling pathway molecules in different groups. ^∗^: significant compared to group (C) (*P* < 0.05); #: significant compared to group (M) (*P* < 0.05).

These changes in signaling molecules were further confirmed by immunohistochemistry analysis. As shown in Figure 7, among all the tested PI3K-AKT pathway molecules, VCD mainly induced a significant decrease of AKT phosphorylation (Figure 7(c), (*P* < 0.05)) and a significant increase of BAX (Figure 7(e), (*P* < 0.05)) and cleaved caspase-3 (Figure 7(f), (*P* < 0.05)) expression in rat ovaries. High-dose of GNC significantly increased the AKT phosphorylation level and decreased the expression of BAX and cleaved caspase-3 (Figures 7(c), 7(e), and 7(f)). But low-dose of GNC did not show significant therapeutic effects on the regulation of the PI3K-AKT signaling pathway.

## 5. Discussion

DOR is one of the main causes of poor infertility outcomes and a major challenge even for patients who turned to assisted reproductive therapy for help. Although hormone replacement therapy used by Western medicine can ameliorate the clinical symptoms such as amenorrhea to some extent, the actual ovarian reserve is not fundamentally improved. Also, there exist side effects of reproductive hormone application. Traditional Chinese medicine has a special strength in treating female reproductive problems. As a long used multiherbal formula, GNC has been proved to be effective in treating a series of menopause and aging-related diseases [4, 5, 8, 23]. Based on preliminary clinical application and the speculation that DOR can be considered for the acceleration of ovarian reserve decline ahead of age, we brought up the assumption that GNC should be effective in treating DOR. In this study, by using a DOR rat model induced by VCD, we have validated that GNC is effective in protecting ovarian reserve by activating the AKT signaling pathway and decreased cellular apoptosis.

Decreased number and quality of available follicles are major characteristics of DOR. Both our present results (Figure 2) and a previous report [15] have confirmed that VCD can specifically induce the depletion of primordial and primary follicles without affecting other important organs [24]. This delicately replicates the initial stage of DOR and makes VCD a good agent to induce a DOR model than other approaches to establish DOR models like chemotherapy drugs. One of the important clinical abnormalities of DOR patients is the elevated serum FSH level. However, since VCD only influences follicle numbers of initial stages of follicle stages, the serum FSH level remains unaffected in model rats (data not shown). This may also explain the results that serum *E*_2_ and *T* levels remained unchanged in model rats compared to rats in the control group (Figure 3). But we found a significantly decreased FSHR protein expression in western blot and immunohistochemistry analysis (Figures 4(d) and 5(d)). This indicates a decreased ovarian response to FSH stimulation in model rats. MVH is a specific marker of germ cells expressed in the oocyte of follicles at various stages [25]. AMH is mainly secreted by granulosa cells of primary follicles, secondary follicles, and small antral follicles and has been widely applied in the clinic as a good marker to predict ovarian reserve because it does not fluctuate with the menstrual cycle [26]. We selected these two molecules as biomarkers of follicles at the early stages to validate the effects of VCD. Also, we found significantly downregulated MVH and AMH protein expression in the ovaries of model rats (Figures 4(b) and 4(c) and Figures 5(b) and 5(c)), thus confirming the effectiveness of the VCD-induced DOR model.

Based on the model, the effects of GNC were revealed by the increased ovarian index and the increased number of primordial and primary follicles (Figures 2(a) and 2(b)). Also, surprisingly, the antral follicle count was also significantly improved compared to the control group by high-dose of GNC. This effect may indicate that GNC not only improves follicle development in the early stages but also promotes antral follicle development. MVH, AMH, and FSHR expression levels were also found to be increased as high as normal rats in high-dose of the GNC group. These results confirmed that GNC can improve the ovarian reserve of the VCD-induced DOR rat model and improve ovarian responsiveness to FSH stimulation, although only high-dose of GNC was found to be effective enough. Since traditional Chinese medicine puts much emphasis on the prevention of disease, we started GNC treatment at the same time as VCD administration. This raises two possibilities for the increase of primordial follicle and primary follicle numbers. The first possibility is that GNC prevented the damage caused by VCD, thus prevented the loss of follicles in the first place. This is the possibility we further validated. The second possibility is that GNC increased the formation of primordial and primary follicles after the VCD damage. Traditional dogma holds that the ovarian follicle pool is fixed after birth. The number of follicles will only decrease with the increase of age. However, recent studies have raised the possibility of the existence of ovarian germline stem cells [27, 28] or the formation of primordial follicles by bone-marrow-originated stem cells [29, 30]. Also, the other traditional Chinese herbal medicine study has also found similar results of improved MVH expression, indicating the increase of primordial follicle numbers [31]. Whether the effects of GNC involve the participation of stem cells remains to be studied.

According to previous reports [17], VCD inhibits the phosphorylation and nuclear localization of AKT in the oocyte of primordial and small primary follicles. Also, one of the canonical effectors of FSHR is the PI3K-AKT pathway. To figure out whether GNC prevents this inhibition effect by improving FSHR-related AKT signaling activation and further reduced ovarian cell apoptosis, we analyzed the protein expression of several key targets of the PI3K-AKT signaling pathway by western blot and immunohistochemistry. We found that VCD significantly inhibited the p-AKT expression, decreased FOXO3a level, and increased ovarian proapoptosis molecules BAX, cleaved caspase-3, and PARP expression, while high-dose of GNC significantly promoted AKT phosphorylation, increased FOXO3a level, and decreased the above proapoptosis molecules (Figures 6 and 7). It is worth noticing that PI3K-AKT signaling is a critical signaling pathway in both granulosa cells and oocytes. In granulosa cells, it is related to cell proliferation, apoptosis, and steroidogenesis [32]. This AKT-activation effect is beneficial for both oocyte and granulosa cells to promote follicle maturation.

Except for gonadotrophins, estrogen is also an essential hormone for the regulation of follicle development. Cellular responses to estrogens are mediated by ER*α* and ER*β*. In this study, we found neither VCD nor GNC exerts a significant effect on ER*α* expression. But VCD significantly decreased ER*β* expression in the ovaries, and high-dose of GNC recovered ER*β* protein level. This is consistent with our previous finding that GNC is capable of increasing the expression of ER*β* in the hypothalamus of ovariectomized rats [33]. As a nuclear receptor, it has been well recognized in an ER*β*-mutant rat model study that DNA binding-dependent transcriptional function of ER*β* is critical for preovulatory follicle maturation and ovulation [34]. But extranuclear signals including the PI3K-AKT pathway have been reported to be activated by ER*β* in brain disease studies [35, 36] and myocardial studies [37]. The effect of nontranscriptional activation of ER*β* remains unknown in ovarian researches. Therefore, whether the effect of AKT-activation involves the increased ER*β* activity requires further study.

In summary, GNC improves ovarian reserve in VCD-induced DOR rats. The underlying mechanism was possibly through the inhibition of cell apoptosis and primary follicle atresia through the activation of the AKT signaling pathway. Whether GNC acts on the AKT signaling pathway directly by stimulating FSHR or ER*β* needs further verification by in vitro experiments.

## Figures and Tables

**Table 1:** The product information of the primary antibodies used in this study.

Target	Catalog number and product source	Application^∗^ (dilution rate)
DDX4/mouse vasa homolog (MVH)	ab13840, Abcam, USA	WB (1 : 1000); IHC (1 : 200)
Anti-Müllerian hormone (AMH)	LS-B4020, Lifespan Biological Sscience Co., USA	WB (1 : 1000); IHC (1 : 100)
Follicle-stimulating hormone receptor (FSHR)	NBP1-46302, NOVUS, USA	WB (1 : 1000); IHC (1 : 250)
Estrogen receptor *α* (ER*α*)	ab32063, Abcam, USA	WB (1 : 1000); IHC (1 : 200)
Estrogen receptor beta (ER*β*)	ab3577, Abcam, USA	WB (1 : 1000); IHC (1 : 500)
Forkhead box O3(FOXO3a)	#12829, CST, USA	WB (1 : 1000); IHC (1 : 3000)
PolyADP-ribose polymerase (PARP)	#9532, CST, USA	WB (1 : 1000);
Phosphatase and tensin homolog deleted on chromosome ten (PTEN)	#9188, CST, USA	WB (1 : 1000); IHC (1 : 125)
Phospho-AKT (p-AKT, Ser473)	#4060, CST, USA	WB (1 : 2000); IHC (1 : 100)
AKT	#2920, CST, USA	WB (1 : 2000); IHC (1 : 250)
BAX	#14796, CST, USA	WB (1 : 1000); IHC (1 : 400)
BAD	#9239, CST, USA	WB (1 : 1000)
Cleaved caspase-3 (Asp175)	#9664, CST, USA	WB (1 : 1000); IHC (1 : 2000)

^∗^WB: western blot; IHC: immunohistochemistry.

## Data Availability

All data used to support the findings of this study are available from the corresponding author upon request.
